# Next‐level multiomics helps define new critical pathways in developmental hematopoiesis

**DOI:** 10.1002/hem3.42

**Published:** 2024-02-01

**Authors:** David G. Kent

**Affiliations:** ^1^ Department of Biology, York Biomedical Research Institute University of York York UK

After nearly a decade of large‐scale single‐cell multiomic studies involving epigenome, transcriptome, and proteome profiling, researchers are now beginning to move toward understanding the role of endogenous small molecules that help shape the cellular response(s) in a more dynamic fashion. While a number of early studies have focused on generating first‐pass data sets showing what sorts of molecules might be present and might potentially influence cell decision‐making, very few studies go into trying to understand the specific roles of endogenous small molecules in regulating specific processes, and the field is in urgent need of more detailed mechanistic studies.

Last year a research paper in *Nature Communications* emerged from Emery Bresnick's group, which started to ask these probing questions in the context of erythropoiesis. The study by Liao et al.^1^ used lipidomics to add critical new information to the puzzle that is “cell fate transition” during developmental erythropoiesis and revealed a critical new role for ceramide homeostasis in regulating erythropoietin (EPO) signaling in cells.

Ceramide is a sphingolipid that has been documented to play general roles in both cellular growth and cell survival and higher levels are often observed in states of cellular stress.^2^ While it does not typically have high expression levels in the plasma membrane of cells, it is upregulated upon cell stimulation and has been hypothesized by a number of research groups to be a key regulator of lipid raft structure and stability.^3^ Lipid rafts are already known to play major roles in cytokine signaling and the regulation of cellular fate, but tools and approaches to study these processes and relationships in a dynamic fashion are sorely lacking.

In their paper, Liao et al. first identified sphingolipid metabolism as a GATA binding protein 1 (GATA1)‐regulated process and utilized their GATA‐1‐null erythroid precursor cell complementation system to identify specific components of the sphingolipid metabolic pathways.^1^ They specifically identified Degs1 as a modulated factor and then undertook a lipidomic screen using different ceramide intermediates to show that GATA1 plays a regulatory role in ceramide homeostasis. Pharmacological inhibition of sphingolipid delta(4)‐desaturase (DES1) (the gene product of Degs1) was then shown to specifically deplete fetal erythroblast growth in culture compared to myeloid progenitors that tolerated DES1 inhibition. This finding was further extended to the specific cytokines (stem cell factor [SCF] and EPO) that drive erythroblast function, and it was shown that DES1 inhibition in human erythroblasts did not impact the expression of proto‐oncogene c (KIT) (SCF receptor) or EPO receptor (EPOR) but did impact the phosphorylation of downstream targets, thus leading the authors to speculate that ceramides influence the downstream signaling of cytokines, a process they termed “ceramide homeostasis‐dependent commissioning of signalling.” They conclude the paper with a series of additional experiments that try to pinpoint where ceramide imposes its regulatory role and identify a “dual component mechanism” whereby SCF and EPO signaling were regulated at different stages (with EPO/EPOR/Janus Kinase 2 activation being altered but SCF/KIT signaling being regulated via a postreceptor mechanism). As all of these latter studies had to be worked out in modular in vitro systems, there remains substantial work to identify the exact role of specific molecules in patient cells and where we might intervene therapeutically.

These findings, in combination with earlier work in other hematopoietic cells, including the 2019 work of Xie et al., which identified DEGS1 inhibition as a modulator of CD34^+^ hematopoietic stem cell self‐renewal,^4^ should prompt a number of researchers to consider the role of endogenous small molecules such as lipids in the key cellular processes that regulate the fate of hematopoietic cells. Since a large majority of stem and progenitor cells are directly regulated by cytokine signaling, this paper not only gives us new insight into the processes that govern cell state transitions during development, but it also paves the way for labs across the world to consider expanding their own multi‐omic approaches to include lipid and metabolite profiling. If bioactive lipids are more generally driving cytokine:transcription factor‐dependent regulatory networks, then this paper is only the beginning of an incredibly important process for just about every cell state transition in hematopoiesis and during malignancy.

## AUTHOR CONTRIBUTIONS

David Kent is the sole author of this work.

## CONFLICT OF INTEREST STATEMENT

The author declare no conflict of interest.

## FUNDING

The Kent lab is funded by the UK Medical Research Council, the BIll and Melinda Gates Foundation, and Cancer Research UK.

## Data Availability

Data sharing is not applicable to this article as no new data were created or analyzed in this study.
